# Acute exacerbation of interstitial pneumonia associated with rheumatoid arthritis during the course of treatment for *Pneumocystis jirovecii* pneumonia: a case report

**DOI:** 10.1186/s13104-016-2052-0

**Published:** 2016-04-26

**Authors:** Takeshi Kuroda, Hiroyuki Takeuchi, Yukiko Nozawa, Hiroe Sato, Takeshi Nakatsue, Yoko Wada, Hiroshi Moriyama, Masaaki Nakano, Ichiei Narita

**Affiliations:** Division of Clinical Nephrology and Rheumatology, Niigata University Graduate School of Medical and Dental Sciences, 1-757 Asahimachi-Dori, Chuoku, Niigata City, 951-8510 Japan; Department of Respiratory Medicine and Infectious Diseases, Niigata University Graduate School of Medical and Dental Sciences, 1-757 Asahimachi-Dori, Chuoku, Niigata City, 951-8510 Japan; Department of Medical Technology, School of Health Sciences, Faculty of Medicine, Niigata University, 2-746 Asahimachi-Dori, Chuoku, Niigata City, Niigata 951-8518 Japan

**Keywords:** Rheumatoid arthritis, Etanercept, *Pneumocystis jirovecii* pneumonia, Architectural distortions, Case report

## Abstract

**Background:**

*Pneumocystis jirovecii* pneumonia (PCP) is potentially fatal infectious complication in patients with rheumatoid arthritis (RA) during immunosuppressive therapy. Hospital survival due to human immunodeficiency virus—unrelated PCP reaches to 60 %. The high mortality rate results from difficulties in establishing an early diagnosis, concurrent use of prophylactic drugs, possible bacterial coinfection. We herein report a case of PCP in RA patients who developed the architectural distortions of lung in spite of combined modality therapy.

**Case presentation:**

A 73-year-old Japanese woman with RA was admitted with shortness of breath. Five weeks previously, she had been started on etanercept in addition to methotrexate (MTX). Chest computed tomography (CT) demonstrated diffuse ground glass opacities distributed throughout the bilateral middle to lower lung fields, and serum β-d-glucan was elevated. Bronchoalveolar lavage fluid revealed no *P. jirovecii*, but the organism was detected by polymerase chain reaction method. Trimethoprim/sulfamethoxazole was administered with methylprednisolone pulse therapy. However, the follow-up chest X-ray and chest CT demonstrated aggravation of the pneumonia with architectural distortions. Additional direct hemoperfusion with polymyxin B-immobilized fibers and intravenous cyclophosphamide therapy were insufficiently effective, and the patient died on day 25.

**Conclusion:**

The architectural distortions of lung should be considered as a cause of death of PCP. For this reason, a high suspicion of this infectious complication must be kept in mind in order to establish an early diagnosis and treatment in patients with RA managed with MTX and biologics.

## Background

Pneumonia is among the most frequent potentially fatal infectious complication in patients who are immunocompromised. The incidence of *Pneumocystis jirovecii* pneumonia (PCP) is estimated to be 0.2–0.5 % in patients with rheumatoid arthritis (RA) undergoing therapy with biologics in Japan [1]. The most frequent symptoms are progressive dyspnea, dry cough and a low-grade fever [2]. Interstitial lung disease (ILD) is a well-established and debilitating extra-articular manifestation of RA. ILD is the primary form of pulmonary involvement in RA, with a prevalence ranging from 4 to 68 %, mostly in the 50–60-year age group [3]. Respiratory symptoms may precede the onset of articular symptoms in 10–20 % of cases [4]. Pulmonary fibrosis including multiple thin-walled cysts, which varies in occurrence and severity, is radiographically evident in 10–34 % with PCP patients [5]. In patients with acquired immune deficiency syndrome (AIDS), morphological changes at the late stage of infection have been frequently reported. However, there have been few reports on such late morphological changes in non- human immunodeficiency virus (HIV) patients [1, 6–9]. The most common pattern in the early phase of the lung disease is wide distribution of ground glass opacities (GGOs) with a preference for the apical and central lung regions [1, 3, 6, 10–12]. There are few data about the course, duration and morphological changes in patients with PCP who are not immunocompromised due to HIV. Here we report a case of PCP associated with the use of etanercept in a patient with RA who developed architectural distortions.

## Case report

**Fig. 1:**
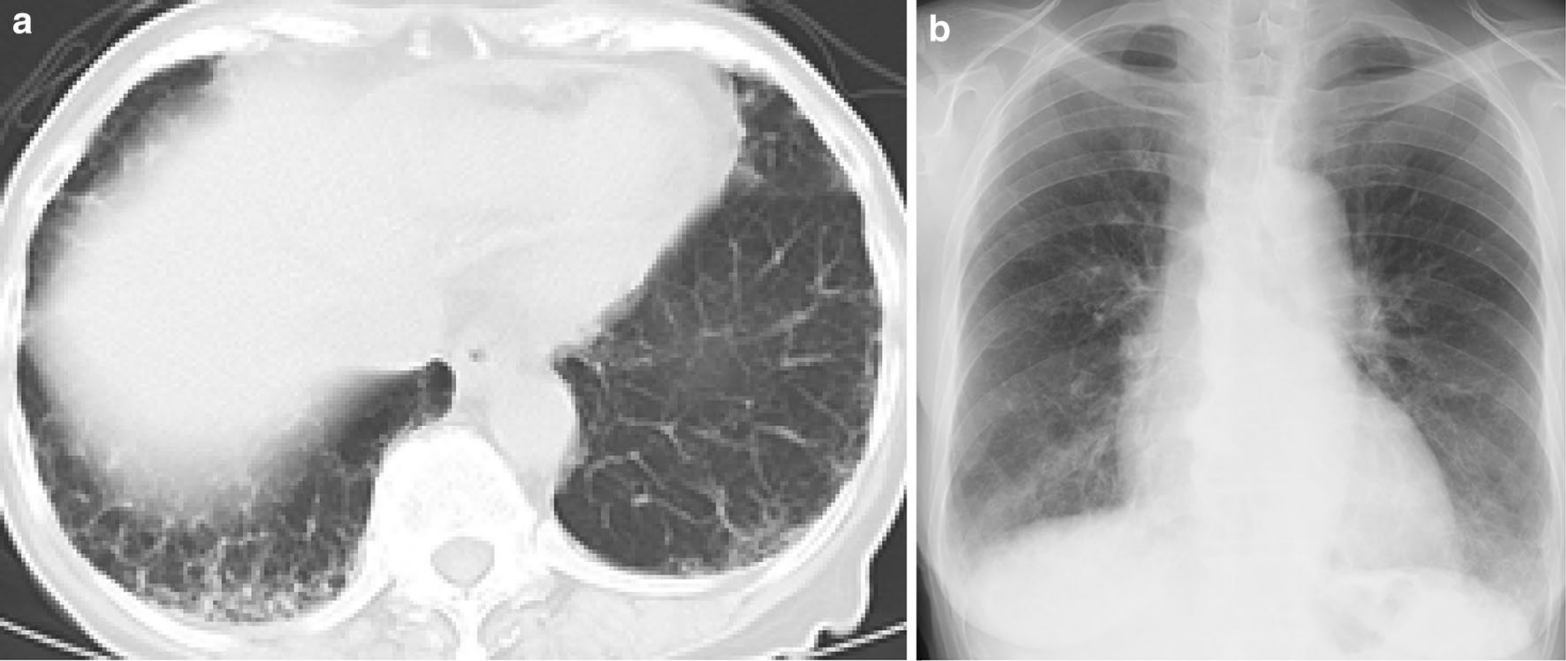
Chest CT and chest X-ray before treatment with biologics. **a** Chest CT was performed 10 month before admission. Mild reticular shadow was observed in the bilateral lower lung. **b** Chest X-ray was performed 2 month before admission. Mild reticular shadow was observed in the bilateral lower lung again

**Fig. 2:**
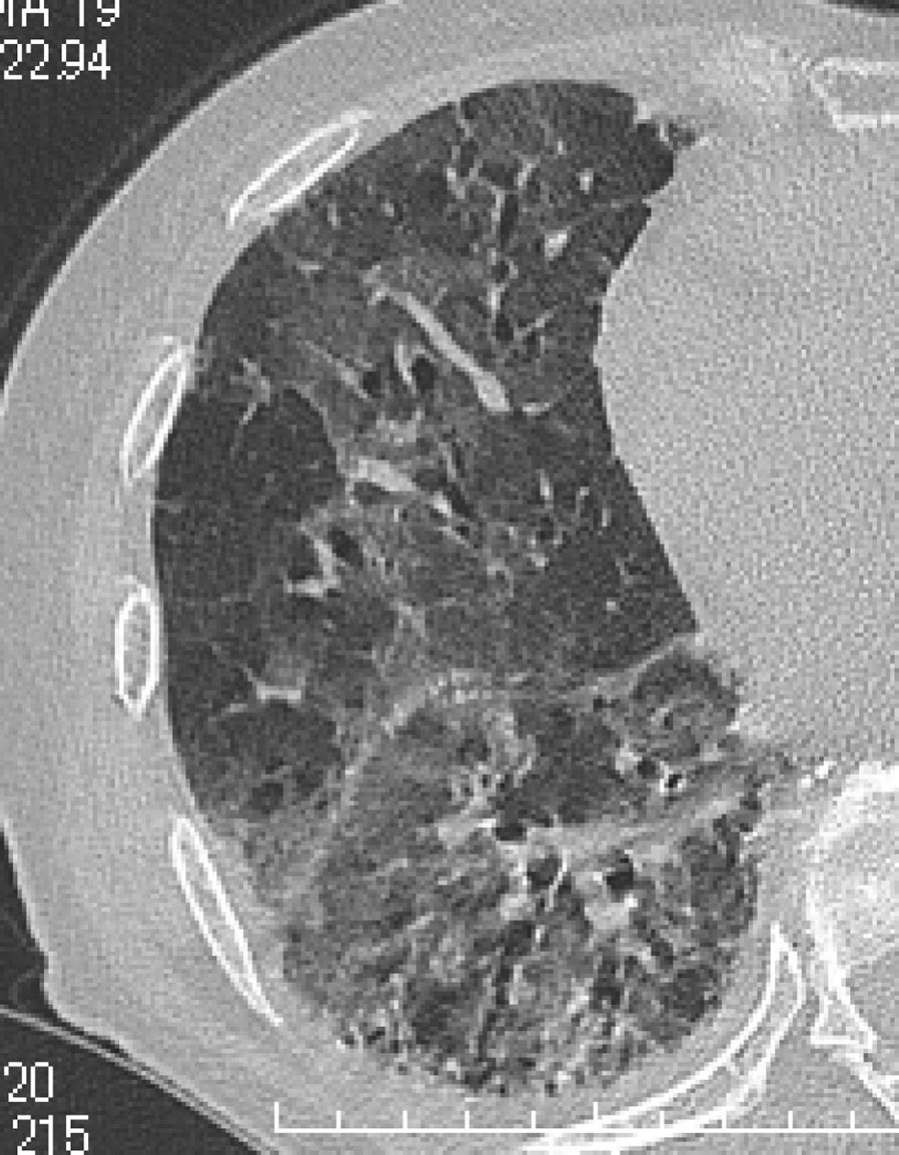
Chest high-resolution CT at the time of admission. A chest high-resolution CT (HRCT) showed GGOs in the lower lung field with thickened interlobular septa and traction bronchiectasis

**Fig. 3:**
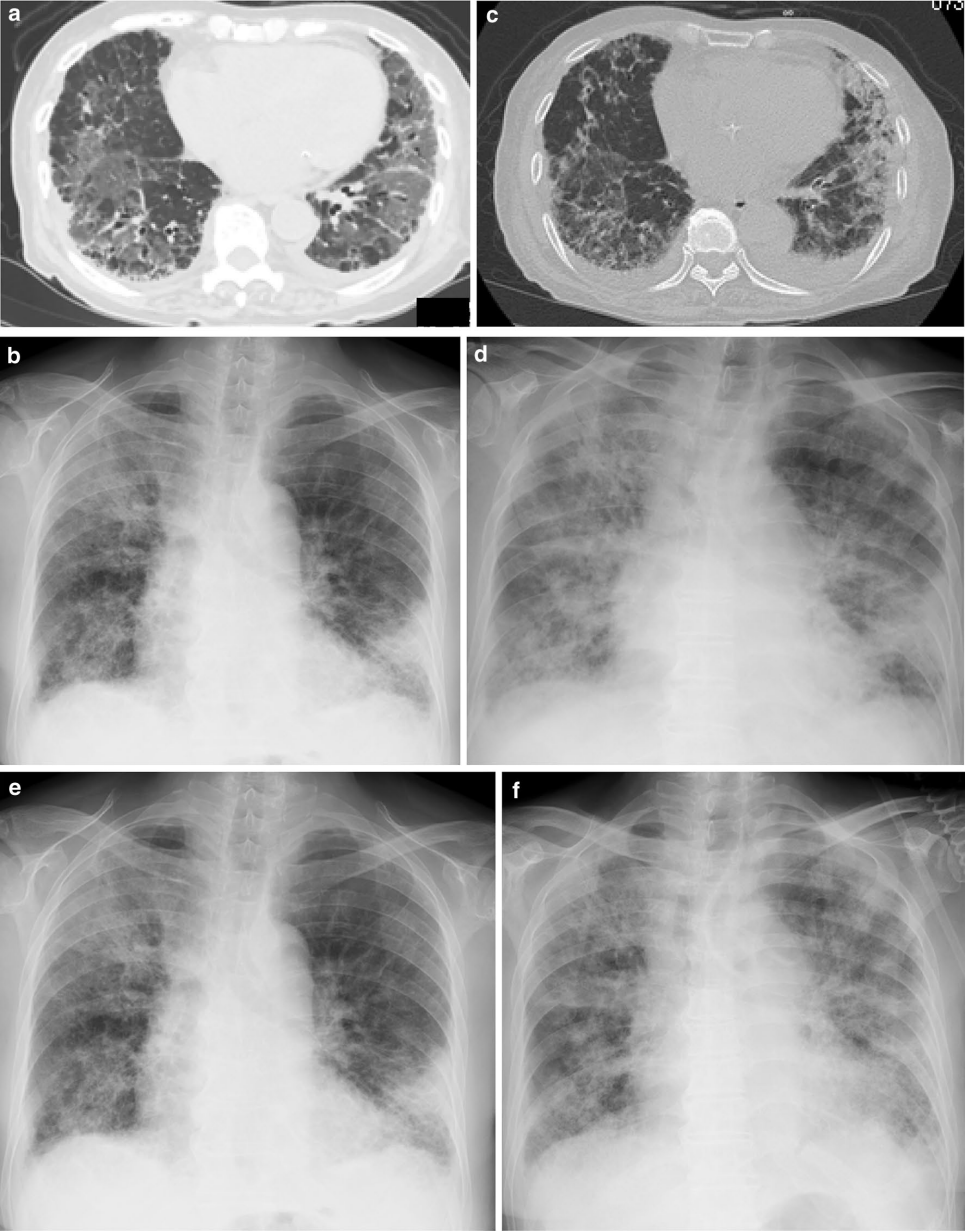
Chest CT and chest X-ray in the clinical course of the patient. **a** Chest CT showed that CT-attenuation of pulmonary infiltrates had increased and the beginning of architectural distortions was evident (second hospital day). **b**, **c** A chest X-ray and CT showed an increase in GGO and parenchymal consolidations with progression of the architectural distortions and pleural effusion (8th hospital day). **d** A chest X-ray showed an increase in GGO with pleural effusion (15th hospital day). **e** A chest X-ray showed no change in GGO with pleural effusion in spite of direct hemoperfusion using a polymyxin B-immobilized fiber column (18th hospital day). **f** A chest X-ray showed an increase in GGO with pleural effusion in spite of intravenous cyclophosphamide therapy (21st hospital day)

**Fig. 4:**
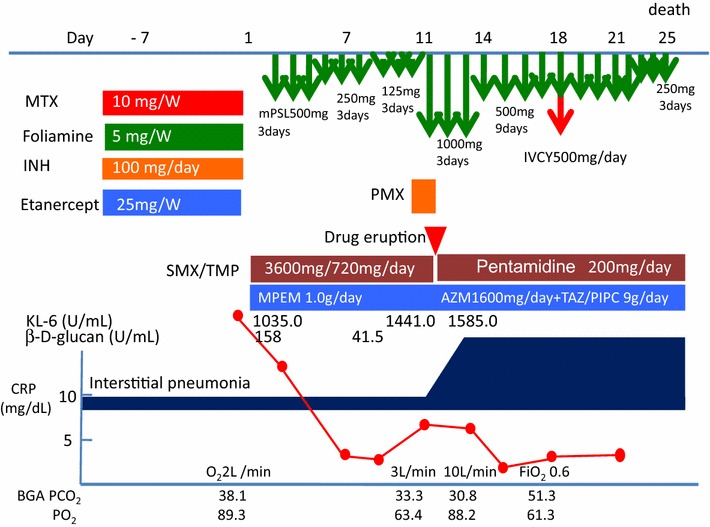
Clinical course of the patient with *Pneumocystis jirovecii* pneumonia. Interstitial pneumonia with architectural distortions was progressed in spite of the treatment with methylprednisolone pulse therapy, direct hemoperfusion with polymyxin B-immobilized fibers and intravenous cyclophosphamide therapy

A 73-year-old Japanese woman had been diagnosed as having RA 5 years before presentation and was initially treated with etanercept at 50 mg/week. Her family history included no consanguinity or collagen diseases. Two years before presentation, she had suffered tuberculous cervical lymphadenitis and had stopped taking etanercept because of remission. After 1 year, she was diagnosed as having interstitial pneumonia by chest computed tomography (CT) (Fig. 1a). Her RA disease activity gradually increased, and she was started on methotrexate (MTX) at 6 mg/week 18 months before presentation. She was referred to our hospital because of her increasing disease activity. Because control of the RA was incomplete, the dose of MTX was gradually increased to 10 mg/week. Two month before treatment of biologics, chest X-ray was performed and diagnosed as having interstitial pneumonia again (Fig. 1b). As the disease was refractory, biological therapy with etanercept at 25 mg/week was readministered when the RA was diagnosed as class 2, stage II. Five weeks later, she presented at our hospital complaining of cough and shortness of breath that had persisted for 2 weeks. On admission, the patient’s body temperature was 35.7 °C and her respiratory rate was 18 breaths/min. Chest auscultation revealed bilateral fine crackles. Arterial blood gas analysis on 2L of O_2_ via a nasal cannula showed a pH of 7.41, PaO_2_ 89.3 Torr, PaCO_2_ 38.1 Torr, and HCO_3_^−^ 23.7 mmol/L. Laboratory examinations revealed a white blood cell count of 6930/μL (neutrophils: 97.0 %, lymphocytes: 3.0 %, monocytes: 0.0 %, eosinophils: 0.0 %, basophils: 0.0 %), lactate dehydrogenase 424 IU/L, C-reactive protein 20.7 mg/dL, procalcitonin 0.83 ng/mL, sialylated carbohydrate antigen Krebs von den Lungen-6 (KL-6) 1035 U/mL, surfactant protein D (SP-D) 262.9 ng/mL, and β-d-glucan 158 U/mL. Although the CT findings did not suggest malignancy, the levels of tumor markers such as carcinoembryonic antigen (CEA), carbohydrate antigen 19-9 (CA19-9), and soluble interleukin-2 receptor (sIL-2R) were elevated at 8.5 ng/mL, 799 and 2954 U/mL, respectively. In addition, an HIV test had a negative result. A chest X-ray showed demonstrated GGOs in the middle and lower lung fields. High-resolution CT (HRCT) showed GGOs with thickened interlobular septa and traction bronchiectasis (Fig. 2). We suspected that the patient had PCP, but MTX-induced lung disease and cytomegalovirus pneumonia were included in the differential diagnosis. We then stopped the MTX therapy and started the patient on trimethoprim/sulfamethoxazole (SMX/TMP) at a TMP dose of 720 mg daily with empirical meropenem antibiotic therapy. On the 2nd hospital day, chest CT was performed again and this showed that CT-attenuation of pulmonary infiltrates had increased and the beginning of architectural distortions was evident (Fig. 3a). Bronchoscopy was performed on the third hospital day. Bronchoalveolar lavage (BAL) performed in the right middle lobe yielded 20 mL of fluid, but no significant pathogens were cultured from it. However, the results of polymerase chain reaction (PCR) for *P. jirovecii* were positive, even though para-aminosalicylic acid and Grocott-Gomori methenamine silver nitrate staining of the BAL fluid revealed no *P. jirovecii* cysts. The patient was diagnosed as having PCP, and SMX/TMP was administered for 14 days. On the 14th hospital day, skin eruption was observed, and this was considered to be a drug reaction; therefore SMX/TMP was switched to pentamidine. On the 14th hospital day, the patient developed dyspnea and her coughing increased. In addition, the PaO_2_ on 3L of O_2_ per cannula decreased to 63.4 Torr. A chest X-ray and HRCT performed on the 8th hospital day showed an increase in GGO and parenchymal consolidations with progression of the architectural distortions and pleural effusion (Fig. 3b, c). These findings were considered to reflect an immune response due to the treatment for PCP with SMX/TMP alone. Therefore, we administered methylprednisolone from 2nd hospital day (500 mg/day intravenously for 3 days, followed by 250 mg/day intravenously for 3 days and 125 mg/day intravenously for 3 days). Shortness of breath was progressive and saturation of O_2_ was decreased, necessitating readministration of methylprednisolone from the 11th hospital day (1000 mg/day intravenously for 3 days and then 500 mg/day intravenously for 3 days). In addition, direct hemoperfusion using a polymyxin B-immobilized fiber column was performed on the 11th hospital day. A chest X-ray performed on the 15th hospital day showed an increase in GGO with pleural effusion (Fig. 3d). The patient’s respiratory condition worsened, and 500 mg of intravenous cyclophosphamide was added on the 18th hospital day. Repeated examination of chest X-ray was performed on the 18th and 21st day and revealed no improvement (Fig. 3e, f). However, The β-d-glucan level was not elevated at any time during the patient’s hospital course. It is known that the level of KL-6, a marker of interstitial lung disease, is increased in patients with PCP. The KL-6 level in our patient was 1441 U/mL on the 10th hospital day and 1585 U/mL on the 11th hospital day. However, the patient’s respiratory status deteriorated rapidly, and she died on the 25th day of hospitalization (Fig. 4).

## Discussion

The incidence of *P. jirovecii* pneumonia (PCP) is estimated to be 0.2–0.5 % in patients with rheumatoid arthritis (RA) undergoing therapy with biologics in Japan [1]. It was indicated that combination therapies with MTX and biologics for RA are a common risk factor for PCP [13]. Advanced age or existing RA lung involvement are also reportedly associated with PCP [11]. Our RA patient who was treated with MTX and etenercept was over 60 years of age and had pre-existing mild interstitial pneumonia and bronchiectasis. Additionally, she was treated with etanercept as well as MTX, but developed PCP only 1 month after the start of etanercept administration. It is possible that the rapid change of immune status after treatment with etanercept and MTX might have led to PCP.

Tasaka et al. [12] have suggested that a diagnosis of PCP should be made based on a β-d-glucan level and positive findings for *P. jirovecii* in BAL fluid on PCR. It has been reported that in non-HIV-infected patients, such as those with RA with PCP, the number of organisms in the airways is relatively (10- to 100-fold) low in comparison with patients who have AIDS-related conditions [11, 12, 14, 15]. In the present case, the diagnosis of PCP was based on elevation the of β-d-glucan level and PCR positivity for *P. jirovecii* in BAL fluid. However, no significant pathogens were cultured from the BAL fluid, and *P. jirovecii* was not detected by Grocott-Gomori methenamine silver nitrate staining.

Upon imaging, the initial appearance of PCP on chest CT was symmetrical, apically distributed GGOs with a perihilar location, a mosaic and consolidations pattern [1, 3, 6, 10–12]. In such cases, the density of infiltrates slowly increases and a mosaic pattern with architectural distortions can evolve from GGOs if appropriate treatment is not provided. Furthermore it has been shown that GGOs rapidly improve after the treatment of specific therapy. Tokuda et al. [16] reported that RA-related PCP shows both diffuse homogeneous GGOs and homogeneous or non-homogeneous GGO with sharp demarcation in the intralobular septa on chest CT. Chest CT in the present case revealed homogeneous GGOs demarcated from the non-affected lung by interlobular septa. Additionally, architectural distortions with irregular linear opacities, reticulations and traction bronchiolectasis were observed, and showed irreversible progression. The reason for these architectural distortions in unclear. With regard to fibrosis, Pareja et al. [17] have suggested several effects of modulation of the host immune response by *P. jirovecii* itself, such as pulmonary immune clearance and other mechanisms. Additionally, it has been not completely clarified that the relationship between anti-inflammatory effects and the gas exchange abnormality, modulation of the host immune response, enhanced of effector function to *P. jirovecii*, enhancement of immune clearance mechanisms of lung. In more than one-third of HIV-negative PCP patients, it has recently been demonstrated that irregular linear opacities, reticulations and traction bronchiectasis were present as architectural distortions. During follow-up for PCP, these signs were reversible completely in more than two-third of them. However, postinfectious fibrosis rarely occurs following PCP. On the other hand, later expansion of lethal interstitial pneumonia despite low levels of serum β-d-glucan may characterize PCP in RA patients who have pre-existing pulmonary lesions. A previous pathological study of HIV-positive PCP patients revealed that the occurrence of fibrosis was variable in autopsy specimens. Either fibroblastic organization of intraalveolar exudates (fibrosing alveolitis) or PCP per se had been reported as causes or promoters of fibrosis [7]. Pentamidine has frequently been used for treatment of PCP, as in the present case, but recently atovaquone has been used for both prophylaxis and treatment. The effects of pulmonary fibrosis after treatment of PCP have not been reported, and further investigations of atovaquone treatment are necessary. It is assumed that the use of adjunctive steroid therapy to reduce the respiratory complications with PCP is beneficial in non-HIV patients [17]. This, with initiation of early diagnosis and the treatment, might explain the rare occurrence of fibrosis and the reversibility of architectural distortions. In the present case, repeated pulse methylprednisolone therapy was performed together with specific antibiotic therapy during the course of disease, but the effects were insufficient. Therefore, direct hemoperfusion using a polymyxin B-immobilized fiber column and intravenous cyclophosphamide therapy were added, because these treatments have sometimes been performed in cases of acute interstitial pneumonia [18]. However, these treatments were not sufficient to arrest the progression of interstitial pneumonia.

Prophylactic treatment for PCP with TMP/SMX should be considered for high-risk patients. However, a recent review considered that it was impossible to predict which patients would benefit the most from TMP/SMZ, and that thousands of subjects would need to be studied before the benefit of TMP/SMZ was proven [19]. TMP/SMZ for prophylaxis might be considered in high-risk patients such as those with interstitial pneumonia and bronchiectasis.

## Conclusions

The PCP is a potentially fatal condition in immunocompromised hosts. The architectural distortions of lung should be considered as a cause of death of PCP. For this reason, a high suspicion of this infectious complication must be kept in mind in order to establish an early diagnosis and treatment in patients with RA managed with MTX and biologics. Further accumulation of similar cases is needed to clarify the etiology and treatment of the architectural distortions.

## Abbreviations

PCP: *Pneumocystis jirovecii* pneumonia
RA: rheumatoid arthritis
AIDS: acquired immune deficiency syndrome
HIV: human immunodeficiency virus
ILD: interstitial lung disease
GGOs: ground glass opacities
CT: computed tomography
MTX: methotrexate
KL-6: sialylated carbohydrate antigen Krebs von den Lungen-6
SP-D: surfactant protein D
CEA: carcinoembryonic antigen
CA19-9: carbohydrate antigen 19-9
sIL-2R: soluble interleukin-2 receptor
HR-CT: high-resolution CT
SMX/TMP: trimethoprim/sulfamethoxazole
BAL: bronchoalveolar lavage
PCR: polymerase chain reaction
